# First reported case of delayed‐type hypersensitivity reaction to non‐hyaluronic acid Polycaprolactone dermal filler following COVID‐19 vaccination: A case report and a review of the literature

**DOI:** 10.1002/ccr3.5343

**Published:** 2022-02-06

**Authors:** Yasamin Kalantari, Afsaneh Sadeghzadeh‐Bazargan, Zeinab Aryanian, Parvaneh Hatami, Azadeh Goodarzi

**Affiliations:** ^1^ Department of Dermatology Razi Hospital Tehran University of Medical Sciences Tehran Iran; ^2^ 48439 Rasool Akram Medical Complex Clinical Research Development Center (RCRDC) School of Medicine Iran University of Medical Sciences Tehran Iran; ^3^ 48439 Autoimmune Bullous Diseases Research Center Tehran University of Medical Sciences Tehran Iran; ^4^ Department of Dermatology Babol University of Medical Sciences Babol Iran; ^5^ 48439 Skin and Stem Cell Research Center Tehran University of Medical Sciences Tehran Iran

**Keywords:** adverse effects, augmentation, case report, coronavirus, cosmetic, cosmetic filler, COVID‐19, COVID‐19 vaccines, delayed‐type reaction, dermal fillers, hyaluronic acid, hyaluronic acid fillers, non‐hyaluronic acid fillers, review, side effects, swelling, vaccination

## Abstract

Cases of filler reactions after COVID‐19 vaccination have been reported. Here, we present the first case of delayed‐type reaction (DTR) to non‐hyaluronic acid Polycaprolactone dermal filler after the second dose of Sinopharm COVID‐19 vaccine which was improved with administration of topical and intralesional steroids.

## Graphical abstract

While rare, cases of filler reactions after COVID‐19 vaccination can occur. Here, we present the first case of delayed‐type reaction (DTR) to non‐hyaluronic acid Polycaprolactone dermal filler after the second dose of Sinopharm COVID‐19 vaccine.

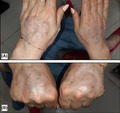

**What do we know?**

- Various adverse effects associated with COVID‐19 vaccines have been reported.
- Cases of filler reactions following COVID‐19 vaccination have been observed in practice.

**What does this article add?**

- While rare, reactions to previously placed Polycaprolactone (PCL) fillers can happen following COVID‐19 vaccinations.
- Individuals seeking both COVID‐19 vaccines namely Sinopharm and PCL fillers should be aware of this phenomenon.

## 1 INTRODUCTION

Dermal filler injections are among the most popular minimally‐invasive cosmetic procedures performed globally.^1^ There exist different types of fillers such as hyaluronic acid (HA), calcium hydroxylapatite, poly‐L‐lactic acid, and collagen‐based fillers.^2^ Polycaprolactone (PCL) derivatives are soft dermal fillers that are biodegradable.^3^ While PCL fillers have a proven safety profile, many adverse effects associated with these fillers such as nodules, granuloma, edema, and bruising have been reported.^4^ Acute type 1 hypersensitivity reactions, which are immunoglobin E (IgE)‐mediated, can occur within minutes or hours after filler injection while delayed‐type reactions (DTR), which are T‐cell mediated, can happen between hours or days after the filler placement.^5^

With the onset of vaccination against the SARS‐CoV‐2 virus (COVID‐19 virus), various adverse effects associated with these vaccines were reported. As well, filler reactions following COVID‐19 vaccination have been observed in practice.^6^ Herein, we present a case of DTR to previously placed PCL filler after getting the Sinopharm vaccine against COVID‐19.

## 2 CASE PRESENTATION

A 62‐year‐old woman without any past medical history or allergies presented to our clinic complaining of multiple nodules with stone‐like firmness in the back of her hands. She had previously received PCL filler within the back of her both hands 2 years ago. She had never experienced any complications with her hand fillers during the past 2 years. Fourteen days after getting the second dose of Sinopharm vaccine, she noticed painless swelling in the dorsum of her hands without any other symptoms. After 2 weeks, she developed multiple hard nodules in the back of her both hands and no extra‐site symptom was noticed. Notably, she did not have any complications after receiving the first dose of Sinopharm vaccine.

After taking a thorough history from our patient, it was proved that she did not suffer from any infection or trauma in that area. Furthermore, she had not started a new medication and she had not undergone any dental or medical procedures between the time of vaccination and symptoms' manifestation.

**FIGURE 1:**
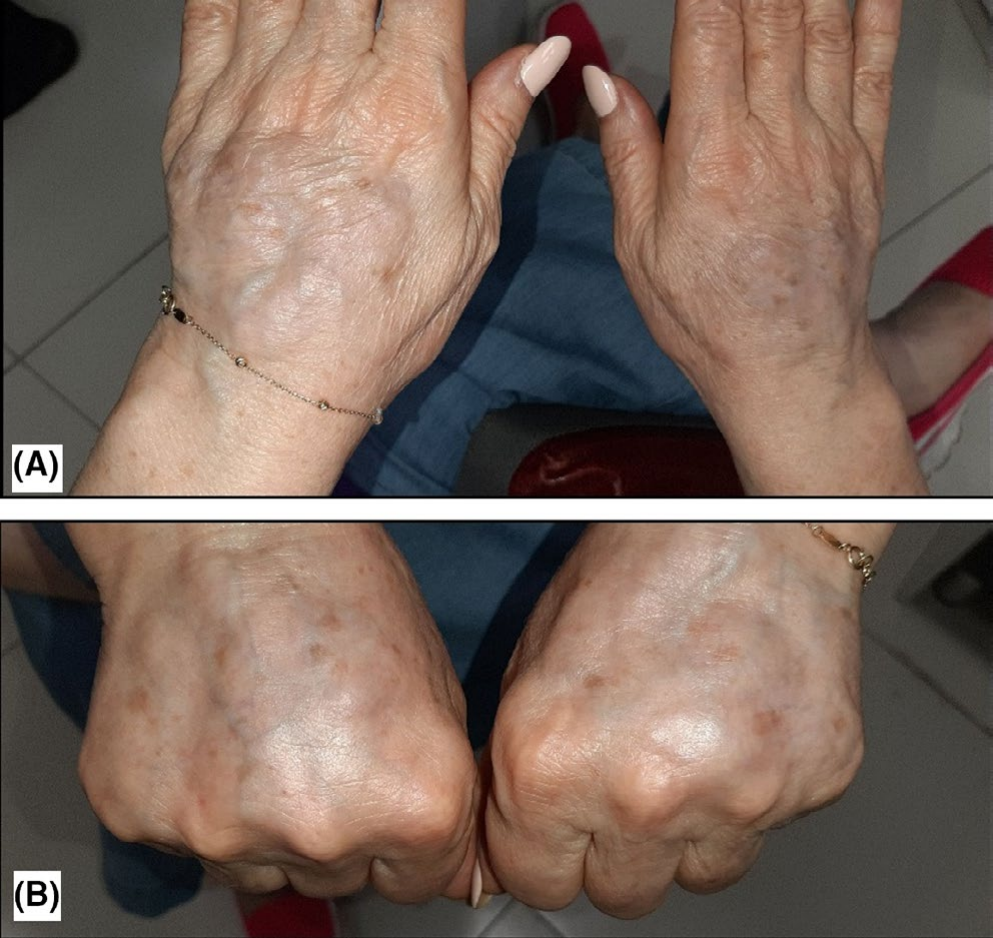
(A and B) Multiple nodules with stone‐like firmness in the back of our patient's both hands in the first visit

Examination revealed multiple nodules with stone‐like firmness in palpation located in the back of her hands (Figure 1). Dorsal hands soft tissue ultrasound imaging showed interstitial edema with low‐level echo fluid without any signs of collection.

**FIGURE 2:**
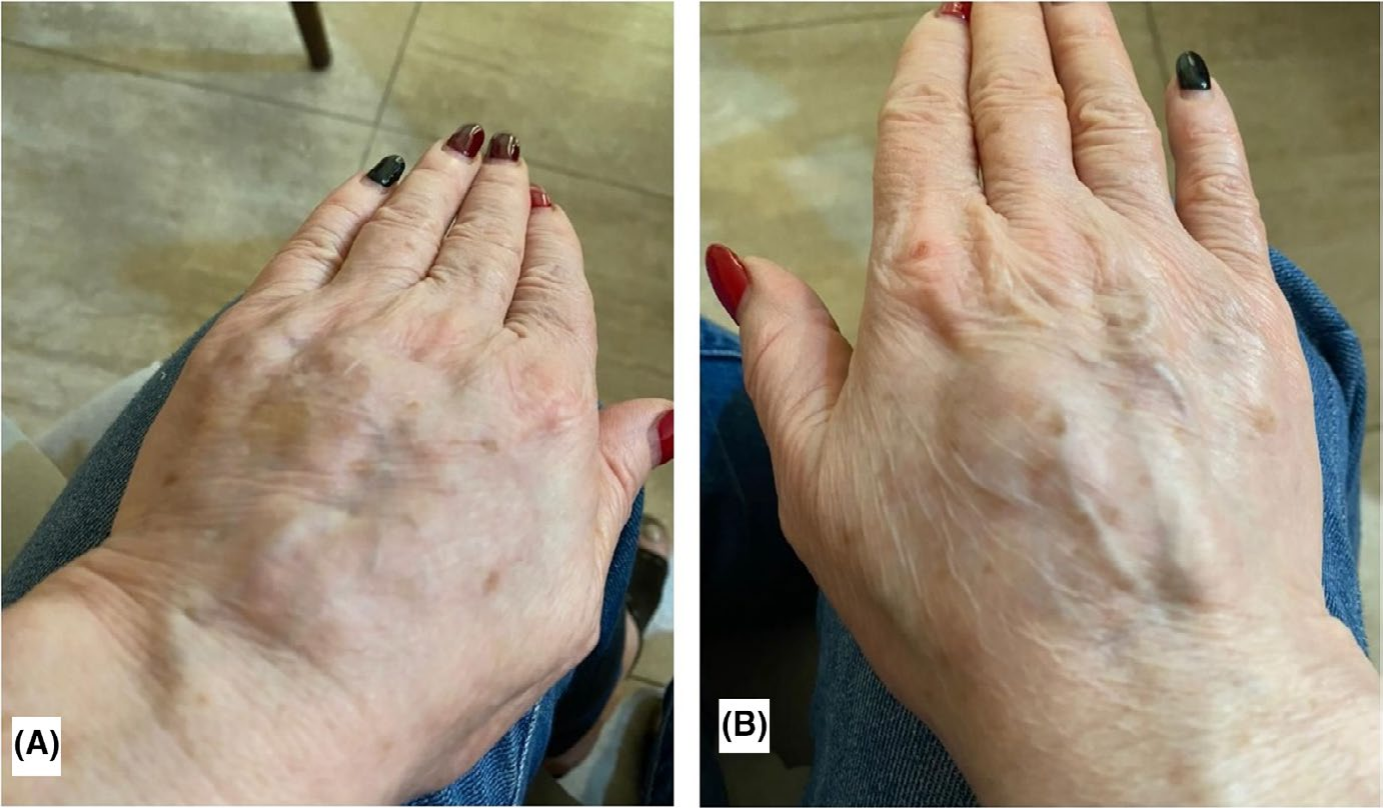
(A and B) Lesions improvement after 4 weeks of treatment

As a result, a DTR reaction following the second dose of COVID‐19 vaccination was made. Single dose of dexamethasone in addition to topical corticosteroid was prescribed. Gradual improvement of lesions was observed after using the topical corticosteroid. Intralesional triamcinolone injection in nodules resulted in a significant improvement. Our patient is still under treatment with topical steroids. During our 4 week follow‐up, no recurrence of the lesions was observed (Figure 2).

## 3 DISCUSSION

Recently, experts defined delayed inflammatory reactions (DIRs) as reactions occurring 2–4 weeks after injections.^6^ The exact mechanisms for DTRs following the filler injection remain unclear, but various factors such as infections, filler properties, trauma, vaccinations, and injection technique are reported to be responsible.^5^ PCL‐based fillers are collagen stimulators stimulating neocollagenesis providing an immediate and temporary filling effect in the target tissue.^4^ In this article, we have provided the first reported case of DTR reaction to PCL filler following Sinopharm vaccination. As well, we reviewed the literature regarding the reported cases of DTR reactions after PCL placement. Furthermore, most reported cases of DTR to dermal fillers following COVID‐19 vaccinations are against HA fillers.

Polycaprolactone‐based fillers are safe and well‐tolerated choices for hand rejuvenation with minimal chance of migration.^7^ In a study done by Figueiredo et al. on 5 women evaluating the efficacy and safety of a PCL‐based filler for dorsal hand rejuvenation using a visual analog scale, satisfaction was rated at 82% at 24 weeks, and patients were 88% likely to re‐treat the same procedure on average.^7^

In a study done by Lin et al. reviewing 1111 treatments with PCL injections, the incidence rate of adverse effects was 4.5% such as bruising/hematoma, swelling, color change, or palpable lumps.^3^ It is worth mentioning that no cases of nodule formation nor granulomas were reported in that study. There has long been a concern about collagen‐stimulating products causing nodules to develop and the injection technique is considered to be one of the most important factors in nodule formation.^3^ In order to prevent nodule development, it has been recommended to use linear threading, fanning, and cross‐hatching techniques in the subcutaneous plane and to avoid injecting more than 0.2 mL.^3^ Histopathology of a nodule shows an overabundance of product, as opposed to granulomas, which reflect an immune origin that depends on the immune status or specific incidents that make alterations in the immune status.^3^ As well, cases of PCL‐induced granuloma have been reported in the literature.

In contrast to HA fillers which can be dissolved by injecting enzymes (hyaluronidase), PCL‐based fillers cannot be instantly removed with enzyme injections. Observation may be the best approach for treating filler‐induced nodules, because most nodules will disappear spontaneously within a year, and short‐term overcorrection might result in deformity.^8^ In comparison, treatment for filler‐induced granulomas consists of observing, massaging, saline or water dilution, intralesional steroids injection, oral antibiotics administration, curettage, and lesion removal.^8^, ^9^, ^10^, ^11^ Moreover, methotrexate has been used in a case of eruptive granuloma following PCL filler placement.^10^ In our case, topical and intralesional steroids were prescribed and a significant improvement of the lesions was observed.

In this article, we performed a brief review of the literature in the Pubmed/MedLine database to address complications including reported granulomas and nodules formation following PCL injection (Table 1). Unfortunately, we were not able to provide a pathologic and histologic assessment in our patient, which is considered as a limitation of our study.

**TABLE 1:** Reported cases of PCL reactions presented as nodule or granuloma formations

Reference number	First author and year of publication	Age and gender	Location	Clinical presentation and type of reaction	Interval between injection and reaction	Management	Outcome
3	Moon et al./2017	36/male	Cheek, nasolabial folds, infraorbital	Granuloma	2 years	Doxycycline 100 mg twice daily for 1 month	Decreased lesions size
9	Skrzypek et al./2019	68/female	Marionette line	Granuloma	13 months	No treatment	No follow‐up
8	Chiang et al./2021	57/female	Tear trough	Granuloma	7 months	Excision	No recurrence
10	Philibert et al./2020	47/female	Cheeks and nasolabial folds	Eruptive granuloma	9 months	Methotrexate, 10 mg per week for 3 months, then 20 mg per week for 9 months	Complete regression
11	Ortiz‐Álvarez et al.	74/female	Nasolabial folds and over both zygomatic arches	Nodules which were then diagnosed with systemic sarcoidosis	3 months	Methotrexate (20 mg subcutaneous weekly) with prednisone (0.17 mg/kg/day)	Significant improvement

## 4 CONCLUSION

Cases of filler reactions after COVID‐19 vaccination have been reported. Notably, most of them were HA‐based fillers reaction to Moderna and Pfizer COVID‐19 vaccines that may well be because HA fillers are by far the most frequently used ones, and the Moderna and Pfizer COVID‐19 vaccines are also most frequently used.^13^ It is worth mentioning that the authors of this manuscript have broadly worked on COVID‐19 and skin as well as cosmetic fields.^14^, ^15^, ^16^, ^17^, ^18^, ^19^, ^20^, ^21^, ^22^, ^23^, ^24^, ^25^, ^26^, ^27^, ^28^, ^29^, ^30^, ^31^, ^32^, ^33^ To the best of our knowledge, this is the first reported case of nodule formation due to the PCL reaction following Sinopharm COVID‐19 vaccination and further investigations with longer follow‐ups are recommended. Our case highlights the importance of reporting similar cases in order to better understand this phenomenon and its management. Of note, individuals and physician should be aware of such reactions in practice.

## CONFLICTS OF INTERESTS

None to declare.

## AUTHOR CONTRIBUTION

All authors contributed to the preparation and finalization of this article. YK and AG contributed to writing the article and study design. ZA and PH contributed to literature review. AS contributed to final editing.

## ETHICAL APPROVAL

This study was approved by the Medical Ethics Committee of the Rasool Akram Medical Complex.

## CONSENT

Written and oral informed consent was obtained from this patient.

## Data Availability

All data used to support the findings of the study are included within the articles.
